# Ugandan health workers’ and mothers’ views and experiences of the quality of maternity care and the use of informal solutions: A qualitative study

**DOI:** 10.1371/journal.pone.0213511

**Published:** 2019-03-11

**Authors:** Susan Munabi-Babigumira, Claire Glenton, Merlin Willcox, Harriet Nabudere

**Affiliations:** 1 Global Health Unit, Division for Health Services, Norwegian Institute of Public Health, Oslo, Norway; 2 Institute of Health and Society, University of Oslo, Oslo, Norway; 3 University of Southampton, Southampton, United Kingdom; 4 Uganda National Health Research Organization, Entebbe, Uganda; Agha Khan University, UNITED REPUBLIC OF TANZANIA

## Abstract

**Introduction:**

Although the coverage of maternity services in some low and middle-income countries (LMIC) has greatly improved, the quality of maternity care remains poor, and maternal mortality rates are high. In this study, we describe the meaning and determinants of maternity care quality from the perspective of health workers and mothers in Uganda, the informal solutions used by health workers to manage their daily challenges, and we suggest ways in which maternal care quality can be improved.

**Methods:**

We conducted a qualitative study in the Mpigi and Rukungiri districts of Uganda. Twenty-eight health workers based at selected health centres participated in structured interviews. Thirty-six mothers, half of whom had delivered at health facilities, participated in focus group discussions. Data were analysed thematically, and informed by the WHO framework on quality of care for maternal and newborn health and by Lipsky’s street level bureaucracy concept.

**Results:**

According to health workers, knowledge of clinical standards and processes, timeliness, and women’s choice during labour, as well as resources, physical infrastructure; collaboration with mothers, professionals and community health workers; were important aspects of good quality care. Mothers’ perceptions of good quality care were largely similar to health workers’ views, though mothers were more concerned about health workers’ interaction skills. Structural challenges sometimes led health workers to develop informal solutions such as asking mothers to purchase their own supplies with variable implications on the quality of care. While several of these informal solutions were useful in addressing bottlenecks in the health system, they sometimes placed additional burdens and personal costs on health workers, created mistrust, inequity in care and negative experiences among mothers who could not afford the extra costs.

**Conclusions:**

Health system structural factors; including technical, interpersonal, resource and infrastructural factors; impede the provision and experience of good quality maternity care at health centres in Uganda. Improving the quality of care will require strategies that address these core problems in the health system structure. Such structural reforms will require political support to commit resources, skilful management and leadership that seek to change organisational behaviour and build trust through good quality, woman-centred maternity care.

## Introduction

In order to achieve universal health coverage, health services, including maternity services, need to be accessible, affordable, and of good quality [1]. Whereas the coverage of maternity services in low and middle-income countries has greatly improved over the last decade, the quality of maternity care remains poor and is linked to persistent high maternal mortality rates [2, 3]. In Uganda, deliveries in health facilities have increased from 37% in 2000 to 74% in 2016, and the number of women attending antenatal care four or more times has increased from 42% to 60% [4, 5]. However, maternal mortality rates remain high in the country, at 336 per 100,000 live births, and suboptimal quality of care has been suggested among the reasons for this occurrence [6]. Improving the quality of maternity care is therefore a policy priority for Uganda [7, 8].

Good quality care has been defined as the ‘extent to which health services to individuals or populations achieve desired health outcomes’ [9]. The health system provides the structure where mothers can access good quality care, and the provision and experience of care are part of the process that determine the quality of care. Good quality care is achieved when healthcare is safe, timely, effective, efficient, equitable, and people-centred [9, 10]. Good quality maternity care is also a human right, and important for equity and dignity of women [11].

Mothers’ experiences of maternity care are partly shaped by the quality of the relationship between health workers and mothers [12]. Health workers’ behaviour, attitudes and skills, and the extent to which health workers provide respectful and competent care determine the quality of this relationship. Trust is vital to the relationship between health workers and mothers [13, 14]. Mothers’ experiences of maternity care can influence their decisions on where to seek care, their demand for maternity services, and ultimately health outcomes for mothers and their babies [15].

In order to provide good quality maternity care, health workers require a well-functioning health system structure that supports evidence-based practice [9]. A well-functioning health system is one where the structural elements; namely the health workforce, service delivery, information systems, medical technologies, financing, leadership and governance; are available, function and interact well [16]. However, health systems are complex, dynamic and resistant to change, making it difficult for managers to build well-functioning health systems [17].

Health workers, as providers of health services and targets of quality improvement programmes, are a key resource at health facilities. Their performance depends on the conditions in the health facility and the broader system in which they work [18, 19], but in several settings, these conditions are less than optimal. As “street level bureaucrats” at the frontline of health services, health workers sometimes make decisions and develop informal or ad hoc solutions to manage the challenges that arise during their work [20]. While these solutions may help fill some of the bottlenecks created by a weak health system, they can interfere with the health workers’ relationship with mothers, create mistrust and worsen inequity in care. In this paper, we describe the meaning and determinants of maternity care quality from the perspective of health workers and mothers in Uganda, the informal solutions used by health workers to manage their daily challenges, and, suggest ways in which maternal care quality can be improved.

## Methods

### Study design

We used an explorative qualitative design to explore the nature of interactions between mothers, health workers and the health system, and other elements underlying these interactions such as the organisation of care, teamwork and staff motivation [21].

### Setting and participants

This study was conducted in July 2014, in the Mpigi and Rukungiri districts of Uganda. We selected Mpigi to represent a well performing district, ranked among the top fifteen well-performing districts in Uganda in two health sector performance assessments [22,23], where local governments were ranked by coverage and quality of services, as well as management indicators. We selected Rukungiri district from among the districts that had improved their performance from below to above average score in the two assessments. However maternal mortality rates are not among the criteria for this performance assessment, and Mpigi district had higher maternal mortality rates than Rukungiri district (Table 1).

**Table 1:** District level demographic data.

	Mpigi District	Rukungiri District
Distance from the capital, Kampala (km)	35	364
Total population [24]	250,548	314,694
Total population of women [24]	122,305	162,624
ANC attendance (% attend 4 times or more) [25]	54.2%	45.9%
Health facility delivery rate in 2014/2015 [25]	69.4%	58.3%
Maternal mortality rate per 100 000 live births [26,27]	198*	117**
Total number of hospitals in the district [28]	1	2
Total number Health Centre Level IV	1	5
Total number Health Centre Level III	25	16
Total number Health Centre Level II	12	66

* Maternal mortality rate for 2013/14 period

** Maternal mortality rate for 2014

### Sampling and data collection

#### Sampling of health facilities

In each district, we, together with the district health officer, purposefully selected one or two facilities to represent the three levels of health centres. Level II health centres provide antenatal care services, level III facilities provide antenatal and normal delivery services, while level IV facilities provide all maternity services including surgery [8]. We were primarily interested in the quality of care at health centres rather than hospitals because health centres, especially in rural Uganda, are often the nearest facility for women seeking skilled care during pregnancy and childbirth. We also sampled public and private-not-for-profit (PNFP) facilities that provided maternity services. Table 2 summarises other characteristics of the facilities included in this study.

**Table 2:** Characteristics of participating health centres.

District	Health centre level (No. of facilities)	Ownership	Maternity services provided	Estimated deliveries per month
Mpigi	Level IV	Public	Full range including surgery	200
	Level III (2)	Public (1), PNFP (1)	Normal deliveries	20–45
	Level II (2)	Public	Normal deliveries	<5 (1), 10–20 (1)
Rukungiri	Level IV (2)	Public	Full range including surgery(1), Normal deliveries (1)	30–45
	Level III (2)	Public	Normal deliveries	20–40
	Level II (2)	Public (1), PNFP (1)	Normal deliveries (1), only ANC (1)	<5 (1), 0 (1)

#### Individual interviews with managers and health workers

One day prior to the interviews, the research team visited each facility to introduce ourselves, request permission to conduct the study, identify participants and schedule interviews. We purposefully selected two or three health workers that worked in the maternity unit, and were available to participate in the study on the day of the visit and their manager. By selecting different cadres of health workers, we aimed to achieve variation in the data. All the twenty-eight health workers that were invited agreed to participate in the study. We conducted in-depth interviews using semi-structured questionnaires that had been pre-tested in Wakiso, a district that did not participate in this study (S1 File: Data collection tools). Interviews were carried out individually so as not to disrupt work, but also to ensure that the health workers were able to speak freely. Questions focused on health workers perceptions of good quality antenatal, intrapartum and postnatal care; factors that influence the provision of this care; and their suggestions to improve the quality of care. Interviews were conducted by one of the authors (SMB, HN), and/or a research assistant, who also took notes and audio recorded the interview. In a few instances, only one of the researchers or a research assistant conducted the interview. Interviews were conducted in English, in a quiet area at the health facility, and lasted 1–1.5 hours. When new ideas were introduced at an interview, we probed for additional information at subsequent interviews, until no new ideas were generated.

#### Focus group discussions with mothers

Each of the participating health centres collaborated with a village health team. A health worker contacted one member of the village health team, and requested their help to identify 6–10 mothers that had delivered a baby six months to one year before our visit. We were unable to ascertain the number of mothers that were invited but declined to participate in the study. We chose to conduct focus group discussions because this was practically easier than meeting each mother at home. In order to encourage unrestricted conversations, we held discussions with only mothers, and meetings were held outside a church or in a community member’s compound.

In each district, we held two focus group discussions (FGD), one for mothers that delivered in a health facility, and another for mothers who had delivered elsewhere, for instance at home with the help of a traditional birth attendant or relative. We included mothers who had delivered elsewhere in order to understand how previous experiences with the health system might have influenced the decision not to have a facility birth. FGDs were conducted in the local language, moderated by one interviewer (SMB or a research assistant) and a research assistant took notes and audio recorded the discussions. Discussions were guided by a selection of open-ended questions that were similar to those used in the interview with health workers. Questions focused on mothers’ experiences of antenatal, intrapartum and postpartum care during their last pregnancy, their perceptions of the available quality of maternity care and how it could be improved. Each FGD lasted approximately 90 minutes.

The audio records of the interviews and FGDs were transcribed in English by the research assistants. We did not return transcripts from the interviews or the FGDs to participants for correction.

### Researcher reflexivity

SMB and HN are female medical doctors that previously worked as clinicians in Uganda but, at the time of the study, were working as health systems researchers. While working as clinicians, they experienced, to varying extents, some of the issues identified in this study and this may have influenced the framing of questions and emphasis placed on themes during analysis. The research team introduced themselves to participants as researchers. Four of the interviewers, one of whom was female, were social scientists with experience in health research in Uganda. Two male interviewers conducted one of the four focus group discussions, and this may have influenced the topics mothers chose to discuss in detail and the depth of data collected.

### Data analysis

The data were analysed using a thematic analysis approach [29] in Microsoft Word 2010. One author (SMB) assigned unique identifiers to the data, read and reread the transcripts to familiarise herself with the data, identify and label themes. The unique identifiers were kept alongside the themes to ease traceability. Other authors likewise read the data to confirm the suggested themes, and identify any additional themes. The authors (SMB, HN, MW, and CG) discussed the themes in order to define their boundaries, identify linkages, and organise them into broad categories. Our data analysis was informed by several theories, including the WHO framework on quality of care for pregnant women and newborns [9], and Lipsky’s street level bureaucrat concept [20]. The WHO framework identifies the health system as the basic structure where the quality of health care is enacted. It identifies the provision of care by health workers and the experience of care by mothers as key components in the process that, together with the human and physical resources, determine the individual and facility level outcomes [9]. Street level bureaucracy theory has been used to explain how frontline workers adapt and develop coping mechanisms to manage the daily realities that arise when implementing policies and, that in the process, may depart from the ideals the service intends to achieve [20].

### Ethics

The Uganda National Council of Science and Technology (SS3436), the Norwegian Ethics Council (2013/2122/rek sør-øst), the District Directors of Medical Services and managers of facilities in participating districts approved the conduct of this study. All participants were informed about the purpose of the study and their rights, including the freedom to withdraw their participation. Participants were assured of confidentiality, and signed a consent form, or used a thumb print to indicate their acceptance to participate in this study. Mothers that participated in the study were given a soft drink and transport refund.

## Results

Twenty eight health workers, 13 from Mpigi district and 15 from Rukungiri district, participated in this study (See Table 3). In addition to providing maternity care, fourteen respondents were managers of the facility or maternity unit. We conducted four focus group discussions (FGD) with 36 mothers. In each district, ten women that had recently delivered at a health facility and eight who did not deliver at a health facility participated in separate focus groups.

**Table 3:** Characteristics of health workers interviewed.

Cadre	Health Centre II	Health centre III	Health Centre IV	Years of experience providing maternity care (range)
Medical Officer	0	0	2	2–3
Enrolled/registered Nurse	4	2	0	1–8
Enrolled/Registered Midwife	2	5	5	1–27
Clinical Officer	1	2	1	1.5–4
Comprehensive Nurse Midwife	0	0	1	11
Psychiatric Nurse	1	0	0	1
Nursing Assistant	1	1	0	0.2, 5
Total Respondents	9	10	9	

Table 4 summarises the categories and themes that emerged from our analysis. While health workers and mothers discussed similar aspects of maternity care quality, they emphasized different aspects. Higher level themes on trust and equity were crosscutting, and are discussed in several categories. Within each category, where available, we present data from health workers and from mothers. We use illustrative quotes and indicate the source using ‘FGD facility’ or ‘FGD non- facility’ for mothers attending facility deliveries or non-facility deliveries respectively.

**Table 4:** Quality of maternity care categories and themes.

Categories	Themes (health workers)	Themes (mothers)
**Technical process of care**	Clinical care process Standards of care Timely care Care that allows women’s choice	Health workers with skills to examine, advise, treat
**Physical resources**	Medicines and supplies Skilled, competent, motivated health workers	Medicines and supplies (available also for neonates) Affordable cost of care Health workers (present, available)
**Environmental conditions and physical infrastructure**	Water, electricity Space Amenities Equipment Blood transfusion infrastructure Transport and communication	Clean, hygienic, well-equipped facilities (e.g. water, electricity, blood transfusion) Amenities (e.g. bathrooms) Facilities that are close, or transport to get to facilities and for referral
**Health worker and mother relationship**	Communication and emotional support	Health workers that treat all women, regardless of social background, with respect
**Professional support and collaboration with communities**	Teamwork Supervision and mentoring Collaboration with village health workers	Reporting systems that respond to complaints
**Higher level factors**	Funding Alignment of policy and practice Geographical location of facilities	

### Technical process of care

#### Clinical care process

Health workers referred to a number of technical processes and clinical services when discussing what they saw as good quality antenatal, intrapartum and postnatal care. For antenatal care, this included health education to help women prepare for childbirth and encourage facility attendance; illness prevention, for instance intermittent presumptive treatment against malaria; screening for infections; and examinations to determine the condition of the mother and baby and identify high risk pregnancies for referral. For intrapartum care, health workers also screened and assessed mothers to determine their capability of a normal delivery, identify the stage of labour, and detect complications in need of referral. Health workers discussed using the partograph to closely monitor the condition of the mother and baby, and viewed it as a useful tool to alert them to intervene. They also described the importance of conducting safe deliveries in clean and sterile environments and the use of techniques such as controlled cord traction for delivery of the placenta. For postnatal care, health workers emphasised immediate baby care, including skin-to-skin contact, keeping baby warm, initiating breastfeeding within the first hour, monitoring baby’s breathing and ensuring that the cord is securely tied; and the close monitoring and assessment of mothers, for instance for bleeding or hypoglycemia. In addition, health workers described health education to promote good cord care, breastfeeding and vaccination (Additional information in S2 File).

When asked to describe good quality maternity care, mothers broadly described the importance of health workers with the skills to examine, advise and treat women.

#### Standards of care

Some health workers also described formalised standards of care when discussing good quality care, including the policy recommendation that women attend at least four antenatal care visits, the postnatal follow-up, and the vaccination schedule. While a few health workers reported using Uganda’s general clinical guidelines [30] which also include maternal health, others reported that they did not have guidelines and relied on knowledge acquired during training and through experience:

‘*For sure we don’t refer to the guidelines*. *We use the knowledge we got from school and experience we are having*. *We don’t have written guidelines on obstetric care*.*‘ (Midwife*, *HCIV)*

#### Timely care

A few health workers described the importance of providing timely care by minimizing mothers’ waiting time or acting promptly to intervene or refer mothers to higher levels of care:

‘*[Good] quality care should start when a mother comes*, *there shouldn’t be a lot of waiting time*. *When a mother comes*, *she should be checked immediately*, *but in most cases this is compromised due to the shortage of human resources*.*’ (Midwife*, *HCIV)*

#### Care that allows women’s choice

One health worker described the importance of allowing women to choose their labouring position:

‘*The mother is positioned*, *made to lie on back or any other comfortable position she [wants]’*. *(Nurse Midwife*, *HCIV)*

### Physical resources

#### Availability of medicines and supplies

When asked to describe the challenges they face when providing maternity care, health workers at all health centres reported that medicines and supplies, including mama kits, were sometimes out of stock. Health workers suggested the supplies received were not aligned to numbers served, leading to stock-outs. In Uganda, the quantity of supplies for health centres at level II and III is determined centrally, also referred to as the ‘push system’, and managers cannot request more according to their need. This is a political decision that is beyond the control of health workers or health system managers.

Another reason for the lack of medicine and supplies was that deliveries take place at Level II facilities even though these facilities are not designated to conduct normal deliveries, but are expected to refer mothers to health centre level III, including stabilising mothers with complications.

‘*One of the biggest challenges is shortage of supplies because for us we are on the push system as Health Centre II …*.*we don’t order what we require*. *During emergencies*, *essential drugs like magnesium sulphate are inadequate all the time*. *We run for months without such supplies*. *[Our request] for these supplies*, *like magnesium*, *disinfectant like jik*, *are not honored [by National Medical Stores]*.*’ (Clinical officer*, *HCII)*

Faced with the lack of supplies, informal solutions were developed. For instance, health facility managers used primary health care funds to buy supplies, or health workers borrowed supplies from other facilities or asked mothers to purchase their own.

Mothers described the availability of medicines and supplies among the aspects of good quality care that attracted them to health facilities. Some mothers expected to find supplies at health facilities, and did not trust health workers’ intentions when asked to bring their own supplies or for money to purchase them. Mothers’ limited financial ability to purchase these supplies sometimes led to non-facility deliveries and could worsen inequity in access to care.

‘*These health workers*, *why do they ask us for gloves and cotton*? *It is a government facility where there are midwives*, *drugs and it is a free health facility*. *We are poor*, *we cannot go to private clinics*.*’ (FGD Facility)*.

‘*The reason I refused to deliver from a health centre is [because] I did not have the supplies required [by the] health facility…… Yes*, *I had planned to deliver at home because I could not afford requirements of a health facility…*. *There are many women who cannot afford to buy gloves*, *polythene*, *and cotton wool*.*’(FGD Non-facility)*

#### The availability of skilled and competent health workers

Both health workers and mothers described the need for sufficient numbers of skilled health workers as part of good quality care. Several health workers described the challenges associated with human resource shortages, for example when doctors or theatre nurses were not available at level IV facilities to provide emergency obstetric care, or when health workers were not able to closely monitor mothers in labour using the partograph:

‘*There is no problem in using the partograph but we don’t have time to [complete] it every 30 minutes … So you find we record after one hour or after one and a half hours*. *We don’t concentrate only on the mother who is in the labour ward’ (Midwife*, *HCIV)*.

When asked how they manage to provide 24-hour care given the staff shortages, health workers reported how they cooperated to cover all shifts. However this resulted in ten to 12-hour, or weeklong shifts; the use of unqualified nursing assistants to provide care; or unstaffed health facilities. The long shifts were sometimes informal arrangements by health workers to allow them extended time off with their families:

‘*Being a hard to reach area* … *we are three midwives and all of us [have families in town]*, *so it is difficult to stay here for a long time*. *So we have agreed to work in shifts*. *We work weekly*, *covering both day and night*. *But this is confidential*, *we have agreed with only the “in-charge”*, *and the district health officer is not aware*. *Midwives have accommodation/ houses with in the health facility*, *the conditions are very difficult because we don’t have water*.*’ (Midwife*, *HC IV)*

Some mothers described how health workers were commonly absent on public holidays, and weekends and this sometimes led to non-facility deliveries:

‘*When labour pains started I went to the health centre but I did not find any nurse because it was a public holiday*. *…I went back home as I did not have transport means to go to hospital*. *I went to a village TBA’s home and she delivered me*.*’ (FGD Non-facility)*

Apart from the shortage of health workers, some health workers indicated how a lack of skills limits their ability to provide good quality maternity care. For instance, some clinical officers, midwives that trained a long time ago, and nursing assistants reported how they lacked skills to use the partograph. Others reported a lack of skills and a need for additional training in managing complicated pregnancies and emergencies such as eclampsia, manual evacuation for post-abortion care, and in quality improvement. Health workers at lower level facilities are expected to recognise symptoms and provide first line management for complicated pregnancies, before referring mothers to higher levels of care. Some mothers reported instances where they believed they were wrongly diagnosed, or had heard about bad experiences they attributed to a lack of skills among health workers:

‘*No*, *she did not [examine me well]*, *and she concluded my baby had no head and she called the hospital to come and pick me because of the assumed complication*. *…*.*I think she did not know much because I produced my baby with a head*.*’ (FGD-Non facility)*

#### Motivated health workers

When we asked health workers to describe conditions they considered important for retaining their job, many referred to sufficient, regular and timely payment of salaries. They considered their present salary insufficient for the work done, for qualifications acquired after employment, or for length of service. Several health workers also discussed how accommodation at the health facility was important, but insufficient for the number of health workers; or in poor condition, with leaking roofs, lacking electricity or water. Other important conditions mentioned were opportunities for career progression, for example through study leave; workshops; professional support through regular supervision, rewards and timely appraisal; as well as a good work environment with sufficient drugs, supplies and human resources.

### Environmental conditions and physical infrastructure

Both health workers and mothers described a clean, hygienic environment as an important aspect of good quality care. A good environment was dependent on the availability of water, electricity, adequate space and amenities (additional information in S3 File). Health workers described how the limited space interfered with women’s privacy and how new programmes such as the involvement of men in prevention of mother-to-child transmission of HIV further stretched the available space. Health workers also reported that lack of amenities such as bathrooms sometimes led to discharge of mothers a few hours after delivery and affected postnatal monitoring of mothers. Health workers also described physical infrastructure; including equipment, blood transfusion, transport and communication facilities; among aspects of good quality maternity care. Mothers emphasised the availability of blood transfusion services and transport to referral facilities among aspects of good quality care. Health workers discussed how the lack of blood transfusion infrastructure challenges with communication and transport sometimes led to delays in referral of mothers to higher-level facilities. In addition, they described a lack of equipment for instance for sterilisation, or for neonatal resuscitation. The lack of equipment, coupled with lack of medicines, supplies and other infrastructural challenges, left health workers feeling frustrated that they could not offer good quality care:

‘*When I came to this facility*, *I was delivering mothers like a TBA because there was no sterilizer for equipment*, *there were no drugs*. *If a mother got a tear*, *there was nothing to repair that tear*. *I tried to get supplies from other health centres but when they were finished I found that I could not [deliver mothers anymore]*. *(Midwife*, *HCII)*.

Health workers described how they developed informal solutions to manage the infrastructural challenges. For instance, when facilities lacked water, health workers sometimes collected water from outdoor water sources, requested water from neighbours, paid for water, or asked mothers to bring their own water. When health workers needed to refer mothers to higher levels of care, they used their own telephone to communicate, collaborated with vehicle owners in the neighbourhood, and sometimes paid for mothers who could not afford the transport costs. Several of these solutions placed additional demands on health workers’ personal resources and time; increased costs for mothers, including paying the health worker for extra help; and left some mothers feeling discriminated against by health workers who seemed to be more helpful to those who could afford the extra costs:

‘*She examines and gives you a referral and that is all*. *She tells you to get out of the health centre lest you bring her problems…*.*It is no longer her problem if you sit in the middle of the road or wherever you want to sit and arrange for your own transport*. *But if you are well off she will let you stay inside the health centre gate*.*’ (FGD Non-facility)*

### Health worker—Mother relationships

#### Communication and emotional support

Both health workers and mothers described the importance of good relationships between them as an important part of good quality care. Health workers used terms such as ‘greet her’ and ‘handle her in a good manner’, to describe what they do when interacting with mothers. They described allowing mothers to drink and to have a companion during labour.

Health workers described how they ‘inform mothers’ or tell mothers what to do as part of good quality care. They tended to emphasise a one-way exchange of information and seldom referred to two-way discussions between health providers and mothers:

‘*She should (be) informed also about labour progress because when you don’t talk to a mother she thinks maybe the midwife is proud*, *but when she is informed she sees that the midwife cares about her*. *Also the relatives need to be informed*.*’ (Nurse Midwife*, *HCIV)*.

Some mothers reported positive experiences and felt cared for by health workers who told them what to do, remained close to the mother during labour, or showed acts of kindness, for instance by making the mother’s bed after delivery. However, some mothers described how they received insufficient information, for instance on reasons for referral. Others felt ignored by health workers who were unavailable and needed to be called repeatedly before they responded to the mothers’ needs. Several mothers described health workers’ communication style as harsh or rude and leaving them feeling uncared for, and this sometimes influenced their willingness to return to the clinic or have a facility delivery:

‘*Some time back I delivered from a government facility*, *but the way they treat us is not good*. *When labour pains have started*, *they shout at you and tell you to shut up*. *Because of that mistreatment*, *I decided to deliver from home the following delivery*. *Not all of the health workers mistreat us but some do*.*’ (FGD Non-facility)*

Some women felt discriminated by health workers because they were considered too young or old for childbearing, unmarried or had few resources:

‘*It is just by chance that you find the security guard at the health centre at night*, *sometimes he is not there*. *And when he tries to wake up the midwife*, *she will first ask him what kind of mother has come*. *If they tell her the mother is not financially okay*, *and she is alone*, *the nurse refuses to come out*. *She knows that mothers who come alone cannot afford to give her money*. *…*..*if she knows there is a man*, *he may give her money*. *They [look down upon] unmarried women*.*’ (FGD Non- facility)*

A few women also described physical abuse by health workers who slapped them:

‘*If they talk and you talk back*, *they beat you…*. *They slap and abuse you…*.*They can even spit on you…*.*’ (FGD Non- facility)*

When probed about mothers’ reports of rude communication, some health workers attributed this behaviour to an individual health worker’s personality, large workloads or norms about accepted ways of communicating in particular communities. Some mothers attributed poor communication to health workers’ workload and lack of training in providing emotional support to mothers.

‘*I think all health providers should be forced to attend a course on handling pregnant mothers*, *because the moment for delivery is almost unbearable*, *and at that time you need to be taken care of*, *but you reach there and nobody cares about you*. *If these health workers cared for us we wouldn’t feel much pain during delivery*.*’ (FGD non-Facility)*

### Professional support and collaboration with communities

#### Teamwork, supervision and mentoring

Some health workers described teamwork and collaboration as an important aspect of good quality care. They appreciated receiving professional support from other health workers at the facility and from higher levels of care, especially when emergencies arose. In addition, some health workers viewed supervision and mentoring as important for ensuring good quality care. While some health workers reported lack of supervision, others reported that they received sufficient supervision and support from district and sub-district managers.

‘*To have good quality care*, *external and internal supervision should be conducted regularly*. *Mentors*, *who are senior staff*, *should help [health workers] adhere to standards’ (Nurse*, *HCIII)*.

#### Collaboration with village health workers

Health workers reported collaborating with village health workers who provide health information, refer pregnant mothers to health facilities for skilled care, and follow up mothers in the community. However mothers did not consider the village health workers as a channel through which their grievances with the health facility could be addressed, and felt ignored or unaware of any actions taken to address their complaints.

### Higher level factors

#### Funding

The Ministry of Finance provides limited funds to the Ministry of Health to implement primary health care activities such as community outreach for vaccination. However, primary health care funds were reported to be insufficient for all of the needs that arise. Health workers therefore felt obliged to develop informal solutions, including the use of their own personal funds, and called for increased funding to improve the quality of maternity care:

‘*PHC [funds] are for immunization*, *outreaches*, *school visits*, *home visits*. *(However*,*) PHC is not enough and the money comes after three months… …*.*so I [sometimes] use my own money’ (Midwife*, *HCII)*.

#### Alignment between planned policy and practice

Some level II health centres included in this study were conducting normal deliveries even though they are not mandated to, and so lacked the equipment and commodities they needed, and struggled to provide maternity care. Health workers described how staffing levels, infrastructure and supplies available at health facilities were insufficient for the large population served, or the increasing range of activities health workers were expected to provide:

‘*Government should change policy of staffing norms [to consider the range of services at health facilities]*.*The system should be reviewed so that they consider space*, *human resources and equipment [in relation to] the number of patients or deliveries’ (midwife*, *HCIV)*.

#### Geographical location of health facilities

We observed that the functionality of health facilities at the same level differed, suggesting that structural issues related to the location of the facility may influence quality of care available at the facility. For instance a health centre level IV in one district had a medical doctor, four midwives, an anaesthetist, a functioning theatre where a caesarean section operation had recently been conducted, and an ambulance. This facility was located close to a tarmacked highway leading to the main urban town, and was only a short distance from the main district hospital. The other health centre level IV in the same district did not have a doctor, but had a clinical officer, midwife and nursing officer, had a theatre that was not in use, and mothers were referred to hospital for caesarean sections. This second facility was located a long distance away from the main urban town, in a hilly area, with difficult access. The location of the first health facility along the highway and related infrastructure such as electricity or easy access to the district hospital, may have made it an attractive place to work and retain health workers.

## Discussion

This study highlights how health workers and women in a low resource setting view and experience the quality of maternity care. Health workers had clear perceptions about the elements they believed constituted good quality maternity care. These included knowledge of clinical standards and processes, timeliness, and women’s choice during labour. Good quality maternity care also included the resources and physical infrastructure needed to provide care; as well as collaboration with professionals and community health workers, and good relationships between health workers and mothers. Mothers’ perceptions of good quality care were in many ways similar to health workers’ views; although mothers tended to focus more on health workers’ interaction skills, and on the resources and infrastructure available at health facilities and how these influenced their access to care than on health workers’ technical competence. Whereas health workers had a clear understanding of what constituted good quality maternity care, they emphasized how limited resources, poor infrastructure and lack of technical skills prevented them from providing this care. These structural challenges sometimes led health workers to develop informal solutions with variable implications on the quality of care. While several of these informal solutions were useful in addressing bottlenecks in the health system, they sometimes placed additional burdens and personal costs on health workers, created mistrust, inequity in care and negative experiences among mothers who could not afford the extra costs.

### Does the available quality of maternity care reflect WHOs standards of care?

The WHO quality of care framework identifies competent and motivated health workers and a well-functioning health system structure as essential for the delivery of good quality maternity care [9]. Our study identified several motivated health workers, and some well-managed facilities that were committed to providing good quality maternity care. However, we also identified a need for additional training of health workers and many health system challenges.

As defined by WHO, the provision of evidence-based routine obstetric care and management of complications are important elements of good quality maternity care. Evidence-based guidelines and partographs are examples of tools that can guide the process and improve the quality of clinical care [30, 31]. Although health workers in this study did not use the terms ‘evidence-based practice or care’, they discussed clinical care processes that are addressed in Ugandan clinical guidelines for obstetric care [32]. However, our findings indicate that obstetric guidelines and partographs were sometimes not available, and the skills, human resources and other resources needed to implement both tools were sometimes lacking. Systematic reviews of barriers to implementation of clinical guidelines [33], and partograph use [34] have indicated similar structural and individual level constraints that interfere with health workers’ adherence to these standards.

The WHO also sees the regular collection, analysis and use of data to assess clinical care processes and monitor health outcomes as a key element of good quality maternity care [9]. Health workers in this study did not discuss this aspect of quality of care. This could be for several reasons, including lack of knowledge of the role of data in quality improvement, lack of data analysis skills, health workers not seeing this as their job, or workloads not giving them enough time to analyse data and reflect on the outcomes of practice. Uganda’s health sector quality improvement framework and strategic plan acknowledges that quality improvement is not yet institutionalised, and proposes the need to strengthen use of data among priority interventions to improve patient safety and quality of health care [35].

According to the WHO, good quality maternity care is a human right with a goal to ensure equity and provide a positive maternal experience through effective communication, respectful, dignified care and emotional support [9]. Although a few women in our study reported positive experiences, several reported disrespectful communication, discriminatory behaviour, lack of emotional support from health workers and undignified care due to poor conditions in health facilities. These negative experiences as well as the high costs of care, particularly among rural poor women, led to some deliveries at home, and could worsen health inequities. Our findings support other research from Uganda that described women’s needs and expectations for timely, clear, and respectful communication as well as emotional support from health workers [36]. The authors reported women’s need for privacy, birth companions and participation in decisions, aspects which were not discussed by mothers in our study. Although women in our study were allowed to have companions, these were often left outside the ward because of limited space and the need to protect other women’s privacy in shared wards. A systematic literature review identified physical or verbal abuse, discrimination, poor rapport between mothers and health workers, poor conditions within health facilities, lack of privacy and denial of birth companions among the negative experiences that constitute mistreatment of women during childbirth [37]. The authors reported that women of lower socioeconomic status were more likely to receive poorer care during childbirth and suggested that mistreatment of women in childbirth erodes trust in the health system, and could influence future decisions on where mothers seek care. Other studies have likewise reported how health care costs can worsen inequity by excluding the poor from accessing health services and create mistrust of the health system [38–41].

### What factors influenced the available care?

Our findings suggest that under-resourced, weak or unsupportive health systems influence the quality of maternity care that health workers can provide [42, 43]. These structural bottlenecks have been linked to suboptimal performance of health workers [18, 19], poor readiness of health facilities to provide emergency obstetric care [44] and avoidable conditions that contribute to maternal and perinatal deaths [45]. Although providing more inputs does not directly lead to good quality care, and a well-resourced facility could provide poor quality care [46], it is undeniable that health workers need essential supplies and infrastructure in order to provide good quality maternity care.

Our findings suggest that the material and financial resources at health facilities were determined at the national level and beyond the control of health facilities managers. For instance supplies and medicines for health centres level II and III were supplied on a “push” system according to national “norms” per level of health centre, regardless of the level of need and the level of use. In addition, funds at health facilities were determined at the national level and insufficient for health system managers to address unplanned-for expenses. Other research has suggested that staffing norms are determined at national level, depend on budgetary allocation for health, and as a result restrict recruitment of additional human resources to health facilities [47]. Uganda spends 7–8% of its gross domestic product on health, and falls short of the 15% target agreed by African Union countries in the Abuja Declaration of 2001 [48].

Our findings also suggest that the shortage of health workers and lack of skills and motivation of available staff sometimes led to suboptimal care. The shortage of health workers led to exhaustion, the use of unqualified nursing assistants to provide care; or unstaffed health facilities, all with consequences for the quality of care available. Another Ugandan study likewise documented shortages of 42%– 70% of nurses and 53–67% of midwives needed at health centres [49], resulting in high workload pressures for the available nurses and midwives, and informal task shifting to nursing assistants. WHO recommends a minimum of 23 health workers per 10 000 population in order to ensure that mothers have access to skilled health workers for delivery [50]. Uganda falls well short of this target; the density of health workers was reduced between 2005 and 2011 due to the rapidly growing population, and lower level health centres were most affected [49,51]. Furthermore, our study provides evidence to support the “inverse care law”, where few health workers are not distributed according to level of need but higher numbers of staff were in more accessible areas [51].

### The use of informal solutions

Our findings demonstrate how frontline health workers sometimes developed informal solutions to manage the bottlenecks in a weak health system. Several of these solutions illustrated health workers’ intrinsic motivation to provide the best care possible, sometimes with a personal cost of time and money. While these solutions may have plugged bottlenecks in the system, they often pushed the burden onto the health worker or the mother and sometimes interfered with the experience of care, for instance when health workers were perceived to provide better care for mothers who could afford the extra cost of care. When health workers asked mothers to purchase their own supplies, this sometimes created mistrust and strained their relationship with mothers who expected to find supplies at health facilities. Health workers were also frustrated about having to borrow supplies from other facilities or pay mothers’ costs of care out of their own meagre resources. Our findings support Lipsky’s Street level bureaucracy theory [20] and demonstrate how frontline health workers, working in a health system with limited resources and large demand for services resulting from policies that promoted health facility deliveries, sometimes developed their own solutions to manage their daily tasks. The health workers appeared to ‘cream off’ and serve mothers who could afford the extra costs of care, and in the process strained their relationships with the mothers who could not afford this care [20]. In addition, our study suggests these solutions can strain health workers’ relationships with the mother and worsen inequities in care. Other research from South Africa likewise demonstrated how nurses at primary care facilities developed coping mechanisms, including categorising patients into those who were eligible or not, as a way to manage the increased workload resulting from the free for service policy but sometimes compromised their professional practice [52].

### What are the implications for practice, policy and future research?

Our findings point to several challenges that could be addressed to improve the quality of maternity care. For instance, at the policy level, our findings suggest the need to review health facility staffing levels, allocated resources and space in relation to the range of maternity services and population served. Doing this would identify areas of greatest need, clarify the capability at health centre level II, and in the process prioritise good quality of care over quantity of health facilities. Campbell et al [53] likewise propose that as part of quality improvement, managers need to re-evaluate maternal health services using data on the location and human resources conducting deliveries, health facility functionality, geographical context and population density in order to determine the level at which comprehensive, basic emergency care and normal deliveries are to be provided. For rural areas with low populations, the authors suggest improving transport arrangements, or providing waiting homes. However, reconceptualising maternity services requires skilled managers and committed leadership that seek to change policies and the way institutions are organised and behave [54]. In addition, managers will require political support to fund these changes.

Our findings suggest the need for managers to increase the use of data to assess and improve the process of care as well as health outcomes. The choice of method would depend on the resources and data sources available, but should target outcomes that are important to end users, such as mothers’ experiences of care, in order to improve the quality of care and health system performance [55]. Managers could also encourage and support use of guidelines through teamwork and collaboration [31], or improve health worker skills and use of partographs through supervision, monitoring, audit and feedback [32]. In addition, managers could improve human resource management, for instance by developing and supervising an effective duty roster, and in the process improve health worker performance, job satisfaction and motivation [19]. Training in management is one strategy that has been used to improve knowledge and skills in planning processes, monitoring and evaluation [56]. However, the evidence base is weak and there is need for evaluation of strategies to improve skills in management of health services.

Our findings also suggest the need to improve relationships between health workers and mothers in order to build women’s trust in maternity services. Other research has likewise suggested that trust is an essential element of health provider mother relationships, and is determined by health workers’ communication, emotional support that demonstrates caring and compassion, mothers’ perceptions of providers’ competence and structural factors that determine access to care [14]. However, there is insufficient evidence on effective strategies to build trust between health providers and their clients [57]. Some scholars suggest that trust between health workers and mothers can be built when health workers are trained to provide interactive two-way dialogue and/or managers regulate health worker behaviour through supervision, appraisal and reward systems as a way to build trustworthy institutions [58]. Rebuilding women’s trust in the health system will also require strategies to overcome other institutional barriers such as workload pressures, and lack of space that interfere with the interaction between mothers and health workers [59].

Our findings also point to the need for more research, in particular studies that can verify the quality of maternity care provided by health workers, and evaluate strategies to improve health worker behaviour. In addition, research on positive deviant cases at health facilities would be useful to guide the development of strategies to further improve the quality of maternity care.

### Strengths and limitations of this study

Our inclusion of different types of health worker cadres at different levels of the health system, as well as mothers who had and had not delivered at a health facility, provide a range of perspectives that increases the trustworthiness of our study. Health workers’ descriptions of what constitutes good quality practice and their descriptions of their own practice may not always reflect actual practice, but may be influenced by social desirability bias and a desire to present themselves in a positive light. We have attempted to address this issue by triangulating this information with mothers’ reports of their experiences. We included only two districts of Uganda and findings can only be generalised to contexts with a similar health system.

## Conclusions

Health system structural factors; including technical, interpersonal, resource and infrastructural factors; impede the provision and experience of good quality maternity care at health centres in Uganda. The enactment of good quality care is further undermined when health workers’ relationship with mothers as well as their time and resources are further stretched by informal solutions that try to plug the bottlenecks created by a weak health system. Improving the quality of maternity care will require strategies that address the structural challenges within the health facilities, at policy and governance levels. These strategies could include reconceptualising maternity services to align available resources to key areas of need, and to improve health worker performance and behaviour through managerial mechanisms such as supervision and monitoring. Such structural reforms necessitate political support to commit resources, skilful management and leadership at all levels of the health system that seek to change organisational behaviour and build trust through good quality, woman-centred maternity care.

## Supporting information

S1 File Data collection tools. (DOC)

S2 File Health workers’ description of the clinical care process for good quality maternity care. (DOCX)

S3 File Health workers’ and mothers’ description of environmental conditions and physical infrastructure at health centres. (DOCX)

## Abbreviations

ANC: Antenatal care
EMONC: Emergency obstetric and neonatal care
FGD: Focus group discussion
HC II: Health centre level II
HC III: Health centre level III
HC IV: Health centre level IV
TBA: Traditional Birth Attendant
WHO: World Health Organisation
