# Supplementary material for: Ugandan health workers’ and mothers’ views and experiences of the quality of maternity care and the use of informal solutions: A qualitative study
Source: PLoS One. 2019 Mar 11;14(3):e0213511. doi: 10.1371/journal.pone.0213511 (PMC6411106; doi:10.1371/journal.pone.0213511)
Supplement: S1 File — (DOC) [file pone.0213511.s001.doc]

**S1 File: Ugandan health workers' and mothers' views and experiences of the quality of maternity care and the use of informal solutions: a qualitative study**

**A) Interview Guide _Health workers /Managers**

*Introduce yourself and thank the respondent for agreeing to the interview.*

State your name; a researcher from Makerere University College of Health Sciences, working on the Skilled Birth Attendants essential requirements project.

**What the study is about:** Skilled attendance at birth is widely recognised as an important strategy that is necessary to reduce the high maternal mortality rates and pregnancy related disabilities in many low-income countries. However, several countries in these settings are yet to achieve the 90% coverage by 2015 target set in the United Nations fifth Millennium Development goal (UN 2011). The need to strengthen health systems in order to attain this and other targets set in the millennium development goals has been emphasised (Freedman 2005, UN 2010).

**Purpose of the study:** To explore the perceptions of health workers and their managers on factors that influence the provision of obstetric care. In particular, we will explore their perceptions on quality of care, challenges in providing this care and priorities for improvement.

You have been selected to participate in this study because you are a health worker (*or a manager of health workers*) whose work involves the provision of obstetric care. You are kindly requested to provide insight based on your official and personal experience in providing obstetric care (*or managing those that provide this care*).

We wish to assure you of utmost confidentiality with the information you provide us during this interview. We request for your signature to confirm consent to participate in this study.

***Name of Health Unit:***

| ***Respondent characteristics***  Current position:  Length of service in that position:  Length of service as obstetric provider:  Professional qualification: |
| --- |
| ***Quality of Care***  What are the acceptable standards for good quality care for a mother and newborn baby before, during and immediately after delivery?  ***You mentioned -------------- (as mentioned by the respondent) can you tell me more about this? (Probing question)***  What challenges do you (*or the health workers you manage*) face when trying to provide good quality care to a mother and newborn babies? What challenges are you facing when using  a) the partograph?  b) clinical guidelines for obstetric care  c) drugs and supplies  d) physical space  Are there additional skills you (*or the health workers you manage*) would like to acquire in order to provide good quality care to a mother in labour and her newborn baby?  If you think back over the last 24 hours, which cadre of health worker has been providing obstetric care? **(Probe morning, afternoon, evening, evening shifts to verify if unit provides 24 hour service)**  In an emergency situation where a caesarean section is required, what professional support for example from an anesthetist, doctors, and theatre nurses is available to you during delivery?  If you think back to the last mother you had to refer to another health centre or hospital (*or referrals handled by this unit*), can you describe this process in terms of  a) referral information b) treatment before referral, c) transport, d) who accompanies the mother,  In general, as regards communication on referral, how do you do this on a regular basis?  In what ways have you (OR *has the facility*) worked with village health teams or lower level facilities to improve the health of expectant mothers and newborn babies?  ***(Probe Supervision, support, education)*** |
| **Employment**  What conditions of service do you consider important in keeping you in your job? (Career path, salary increment, continuing education, management and supervision etc.)  What would you like improved about your conditions of service?  What incentives do you receive for serving in this area? (Allowances, accommodation, transport etc.) |
| **Improving Quality of Care**  If you think back on our discussion, do you have any other suggestions for improving the quality of care for mothers and their babies? |

***Thank you for your time***

**B) Focus Group Discussion Guide _Mothers who did NOT deliver in a health facility**

*Thank participants for coming for the discussion.*

State your name; a researcher from Makerere University College of Health Sciences, working on the Skilled Birth Attendants essential requirements project. This project is looking at what can be done to improve the quality of care for mothers and their babies during delivery. *Emphasize the ground rules (one person speaks at a time, facilitator is in charge of the discussion etc.)*

**Purpose of this discussion:** To explore in depth the perceptions of their experience of the alternative delivery site e.g. traditional birth site during their most recent delivery. This information will be uuseful for decision makers seeking to improve the quality of care for mothers and children in low-income countries.

You have been selected as a key stakeholder and user of health facilities, whose views are important in influencing the quality of care at health centres. You are kindly requested to share your personal experiences of health facilities during your last delivery.

We wish to assure you of utmost **confidentiality** with the information you provide during this interview.

***Note the overall characteristics of the participants of the focus group discussion: all delivered at an alternative site e.g.*** TBA, at least 2 or more children, last born borne in this year.

| ***Participant characteristics***  *Can you please introduce yourself by telling us your name, how many children you have and the place you delivered your most recent baby? By place we mean a health unit or private clinic or somebody’s home. For example, my name is Rita, I have one child, and I delivered her at xx.* |
| --- |
| ***Experience of Quality of Care***   1. Can you describe your experience of the care you received during your last delivery and after? 2. Why did you choose to deliver where you delivered? 3. What do you consider as good quality of care for you and your baby during and after delivery? 4. Was any of you referred to another place during labour? For those who were referred, can you tell us where you were referred and share your experience regarding the process of referral? 5. What suggestions do you have on how quality of care can be improved for mothers and their babies during labour? Probe for suggestions to improve care and not only challenges experienced. |

**C) Focus Group Discussion Guide _Mothers who delivered in a health facility**

*Thank participants for coming for the discussion.*

State your name; a researcher from Makerere University College of Health Sciences, working on the Skilled Birth Attendants essential requirements project. This project is looking at what can be done to improve the quality of care for mothers and their babies during delivery. Emphasize the ground rules (one person speaks at a time, facilitator is in charge of the discussion etc.)

**Purpose of this discussion:** To explore in depth the perceptions of mothers on their experience of the health system during their most recent delivery. This information will be useful for decision makers seeking to improve the quality of care for mothers and their babies in low-income countries.

You have been selected as a key stakeholder and user of health facilities, whose views are important in influencing the quality of care at health centres. You are kindly requested to share your personal experiences of health facilities during your last delivery.

We wish to assure you of utmost **confidentiality** with the information you provide during this interview.

***Note the overall characteristics of the participants of the focus group discussion: all delivered in health unit, at least 2 or more children, last born borne in this year.***

| ***Participant characteristics***  *Can you please introduce yourself by telling us your name, how many children you have and the place you delivered your most recent baby? By place we mean a health unit or private clinic. For example, my name is Rita, I have one child, and I delivered her at xx hospital.* |
| --- |
| ***Experience of Quality of Care***   1. Can you describe your experience of the care you received at during your last delivery and after? 2. Why did you choose to deliver at the health facility? 3. What do you consider as good quality of care for you and your baby during and after delivery? 4. Was any of you referred to another health facility during labour? For those who were referred, can you tell us where you were referred and share your experience regarding the process of referral? 5. What suggestions do you have on how quality of care can be improved for mothers and their babies before, during and after delivery? |
