# Supplementary material for: Ugandan health workers’ and mothers’ views and experiences of the quality of maternity care and the use of informal solutions: A qualitative study
Source: PLoS One. 2019 Mar 11;14(3):e0213511. doi: 10.1371/journal.pone.0213511 (PMC6411106; doi:10.1371/journal.pone.0213511)
Supplement: S2 File — (DOCX) [file pone.0213511.s002.docx]

**S2 File: Health workers’ description of the clinical care process for good quality maternity care**

**Antenatal Care**

Health workers regarded health education and illness prevention as an important part of good quality care during pregnancy. Health education was viewed as important to prepare women for childbirth, and provide information on the importance of regular antenatal care, delivering in health facilities and not at home. As part of illness prevention, health workers also described giving mothers’ vitamin supplements and intermittent presumptive treatment against malaria.

*‘When the mother is pregnant she should [attend] antenatal care…where she is prepared for the coming baby [through] health education. The mother is also [encouraged] to deliver from a health unit, not in the village, during health talks…..The mother also gets the haematinics to take on a daily basis to increase her haemoglobin levels. The mother is given preventive treatment [against malaria] (Nurse Midwife, HCIV)*

Health workers also described screening and assessment of mothers and their unborn babies as part of good quality antenatal care to determine the mothers’ condition, detect high-risk pregnancies needing referral to higher levels of care. Screening and assessment was also important to detect illnesses such as HIV that require counselling and treatment, or identify mothers that have faced domestic violence in order to provide the support they need. Health workers described history taking, measuring parameters such as blood pressure or weight, conducting laboratory tests and diagnostic examinations such as ultrasound scans, among methods used to screen and assess mothers.

*‘If you do your antenatal care well you can tell that this mother has placenta praevia, or abnormal lie and malpresentation, [and refer her in time]’. (Midwife, head maternity unit, HCIII)*

**Intrapartum Care**

Health workers described screening and assessing mothers, as well as monitoring and conducting safe deliveries among components of good quality intrapartum care. Health workers described screening and assessing mothers to diagnose active labour and determine mothers’ capability for a normal delivery. Once mothers were in active labour, health workers described the importance of close monitoring of the progress of labour, and the condition of the mother and her baby in order to determine a mother that would deliver normally or require intervention. They described using the partograph, which they viewed as a useful tool to alert health workers to intervene. Health workers also described the importance of clean and sterile environments when conducting deliveries and using techniques such as controlled cord traction for delivery of the placenta.

*‘During labour, we check blood pressure, pulse, temperature and anaemia. We listen to the fetal [heartbeat]. Check the capability of the mother to deliver; if she cannot deliver, we refer her…. We ensure the environment is clean for safe delivering, [we use] well-sterilized equipment, and we monitor both the mother and baby’.* (Midwife, HCIII)

**‘***We have partographs [and] use them in monitoring mothers in the labor ward. They are good because they help us to know whether this mother is going to deliver with us or will be taken to theatre or be referred.*’ (Midwife, HCIV)

**Postnatal Care**

Health workers regarded health education and illness prevention as an important part of good quality postnatal care. Health education during the postnatal period included the importance of clean cord care, breastfeeding and vaccination. As part of good quality immediate postpartum care, health workers described assessing mothers for tears, bleeding, assessing the baby’s apgar score, birth weight as well as monitoring baby’s breathing and ensuring the cord is securely tied. Health workers also described skin-to-skin contact, keeping baby warm and well clothed, and initiating breastfeeding within the first hour after delivery among aspects of immediate good quality neonatal care. In addition, health workers described the recommended standards for the postpartum period. Health workers described a postpartum clinic visit at 6 days where health workers check that the mother and baby are well, and at 6 weeks to check that the mother is healing well, provide vaccination for the baby, supplementation for the mother when needed, as well as health education.
