# Supplementary material for: Ugandan health workers’ and mothers’ views and experiences of the quality of maternity care and the use of informal solutions: A qualitative study
Source: PLoS One. 2019 Mar 11;14(3):e0213511. doi: 10.1371/journal.pone.0213511 (PMC6411106; doi:10.1371/journal.pone.0213511)
Supplement: S3 File — (DOCX) [file pone.0213511.s003.docx]

**S3 File:** **Health workers’ and mothers’ description of environmental conditions and physical infrastructure at health centres**

***Water and electricity***

Health workers described a clean, hygienic environment as an important aspect of good quality care. A good environment was dependent on the availability of water, electricity, supplies such as disinfectants and sterilising equipment. Although several health facilities were observed to have water tanks for rainwater harvest, health workers reported that lack of water, especially in the dry season, was a challenge for providing good quality maternity care. Health workers developed informal solutions to deal with these challenges. For instance they sometimes collected water from outdoor water sources, requested water from neighbours, paid for water, or asked mothers to bring their own water. Mothers described their experiences when a facility lacked water as follows:

*‘You have to go to the well for [water] to use. If you [are the caretaker] of a mother who has given birth, you have to fetch a jerry can of water because you need to wash clothes and water to bathe the mother. [For mothers without an attendant]...That is what they call suffering. You wait until you are discharged and bathe when you reach home. If you get someone to fetch it for you they would charge you 700/= (0.28USD) per jerry can, because they fetch it from far. And it is a dirty place. You have to step in dirty water and it covers you up to the waist, I think that is why they over-charge us.’ (FGD non- facility)*

Health workers also described how a lack of electricity interfered with their ability to provide good quality care as it created problems when sterilizing equipment, using equipment like oxygen concentrators or using the theatre. The lack of electricity also resulted in extra costs for mothers. While some facilities had solar panels, a few health workers reported how solar energy was insufficient to light the maternity wing, was unreliable during the rainy season, or frequently in need of repair. One health centre level IV lacked a generator, and another had a generator but often lacked fuel.

*‘Another challenge we have is sterilization, we are boiling manually in this era of HIV. We use metallic bowls on a charcoal stove. We have to sterilize everyday’. (Enrolled midwife, HCIII).*

*‘Electricity ‘died’ a long time ago, the moment you reach [the health facility] they ask you to buy paraffin for a lamp. Or they tell you to buy batteries for the torch.’ (FGD Non- facility)*

Mothers also described their need for clean wards in maternity units. A few mothers described experiences of unhygienic conditions:

*‘Someone told me that she was put on the delivery bed where someone else had just delivered from, without cleaning it first… Even the health worker told us that when you come for delivery, buy jik and come with it for cleaning the delivery bed. …so that after you have delivered they clean the place for proper hygiene for the next person to use the delivery bed. (FGD non-Facility)*

***Lack of space***

Several health workers reported how a lack of space interfered with the provision of good quality care. Some activities; for example health education, counselling and immunisation; were outdoors, as was the waiting area. New programmes, such as the involvement of men in prevention of mother-to-child transmission of HIV, stretched the already limited space. Lack of space also meant health workers could not always provide mothers with privacy

*‘Our labour ward is well equipped, we have three delivery beds, but it is too small. I was suggesting to the in-charge that we could get a curtain in the middle of these two beds….There is no privacy when you get to second stage of labour.’ (Midwife, HC III).*

***Lack of amenities***

Health workers also reported how the lack of amenities such as bathrooms influenced the quality of care and sometimes led to early discharge of mothers:

*‘There is no bathroom for mothers.… They have to stay without bathing until they go home. Mothers insist [on quick discharge] so that they can go and bathe. If she delivers in the morning, you keep her up to evening, then you let her go’ (Nurse, HC III)*

***Lack of blood transfusion infrastructure***

Health workers at one level IV health facility reported how lack of blood transfusion infrastructure limits their ability to provide emergency obstetric care:

*‘We have opened a theatre here and we want to provide advanced care for mothers but there are many things that are still missing. There is no ready power, water is still a problem, we don’t yet have enough clothes to use in theatre, or blood to attend to an emergency in case a mother loses a lot of blood.’ (Medical officer, HCIV).*

Mothers also identified the availability of blood transfusion services among aspects of good quality care:

*‘Good service is when there is blood transfusion at the facility where there is delivery service. That is one reason they usually tell us that women are taken or referred to hospital’ (FGD Facility)*

***Lack of equipment***

Several health workers described a lack of equipment, for instance for sterilisation, neonatal resuscitation or beds. When facilities lacked beds, mothers used inappropriate beds for delivery, shared beds or used the floor. The lack of equipment, coupled with lack of medicines, supplies and other infrastructural challenges, left health workers feeling frustrated that they could not offer good quality care:

*‘When I came to this facility, I was delivering mothers like a TBA because there was no sterilizer for equipment, there were no drugs. If a mother got a tear, there was nothing to repair that tear. I tried to get supplies from other health centres but when they were finished I found that I could not [deliver mothers anymore]. (Midwife, HCII).*

***Lack of transport and communication infrastructure***

Health workers described difficulties with communication and transport that sometimes led to delays in referral of mothers to higher-level facilities. One facility received funding for communication from a non-governmental organisation, and one had a radio call system, but it lacked solar panels and was not in use. Health workers described informal solutions to these problems, for instance by paying the cost of using their own telephone, collaborating with vehicle owners in the neighbourhood, and sometimes paying for mothers who could not afford this extra cost:

*‘You can examine a mother and you find that you have to immediately transfer her to the higher health facility but don’t have a standby ambulance. You have to go and look for transport from the neighbours … You look at the mother and she is going to die from your health unit – you [the health worker] have to use your money and transport her immediately to the hospital.’ (Midwife, HCIII)*

Mothers also reported how transport to referral level facilities was a problem that sometimes led to delays that could be risky to the mother and baby. The lack of transport often led to additional costs to mothers including paying the health worker for extra help, and left some mothers feeling discriminated against by health workers who seemed to be more helpful to those who could afford the extra costs:

*‘She examines and gives you a referral and that is all. She tells you to get out of the health centre lest you bring her problems….It is no longer her problem if you sit in the middle of the road or wherever you want to sit and arrange for your own transport. But if you are well off she will let you stay inside the health centre gate.’ (FGD Non-facility)*
